# Reply to Aung et al

**DOI:** 10.1093/cid/ciaa733

**Published:** 2020-06-06

**Authors:** Nicholas J White

**Affiliations:** Mahidol Oxford Tropical Medicine Research Unit, Faculty of Tropical Medicine, Mahidol University, Bangkok, Thailand

To the Editor—Symptomatic malaria is predominantly a disease of childhood in areas of higher transmission (ie, much of sub-Saharan Africa). Most cases of severe malaria occur in children aged <5 years. In these regions, both malaria and sepsis are major causes of childhood death, yet the clinical distinction between the 2 is difficult, particularly if there is no obvious focus of infection [1]. Furthermore, severe malaria predisposes to bacterial infections, particularly with *Salmonella* sp., so a very sick child may have both severe malaria and sepsis. In this epidemiological context, the population prevalence of malaria parasitemia detectable by microscopy or rapid diagnostic tests (RDTs) is high, and on more sensitive testing with polymerase chain reaction assay, a large proportion of the entire population are found to be parasitemic. Thus, when a severely ill febrile child presents to a health facility and the microscopy or RDT are positive for malaria parasites, the admitting clinician is often uncertain. Is this severe malaria, or is it sepsis with an incidental parasitemia, or could it be both severe malaria and sepsis together? Today, an increasing proportion of infections are diagnosed with RDTs, which are not quantitative. With blood film microscopy, finding a malaria parasite count above 10 000/uL is strongly suggestive of malaria as the primary illness in a high-transmission setting [1]. However, the severely ill child could still have both severe malaria and sepsis. In a meta-analysis of 7208 children with severe malaria included in 25 studies across 11 African countries, the mean prevalence of invasive bacterial infections was estimated to be 6.4% (95% confidence interval [CI], 5.81% to 6.98%) [2]. For these reasons, it is recommended that in areas of higher malaria transmission, children suspected of having severe malaria should also receive empirical broad-spectrum antibiotics [1, 3]. In practice, this usually means ceftriaxone.

The situation is different in areas of low malaria transmission. In these areas, it is adults, particularly men, who are more likely to present with severe malaria. The malaria blood smear has greater predictive value, and the diagnosis is therefore easier. In managing adult patients with severe malaria, the World Health Organization (WHO) currently recommends that physicians have a low threshold for starting antibiotics, but that routine empirical treatment with broad-spectrum antibiotics is not necessary [1, 3]. This perspective has been challenged in a recent series from Myanmar. In 2 combined series, 13 (14.9%) of 87 adult patients hospitalized with a positive malaria blood film were bacteremic [4, 5]. It was concluded that “empirical antibiotics may also be appropriate in adults hospitalized with falciparum malaria in low transmission settings, until bacterial infection is excluded” [4]. In contrast, a recent large prospective study of sequential adult patients with strictly defined severe falciparum malaria from Vietnam found only 9 (1.07%) of 845 patients were bacteremic [6]. 

In this issue of *Clinical Infectious Diseases*, the authors of the original series from Myanmar propose that the marked difference between the 2 studies might be explained by unreported antibiotic pretreatment and by blood culture insensitivity, although similar blood volumes were cultured in both studies. As described in Phu et al [7], antibiotic self-treatment was unusual in the Vietnamese patients with severe malaria but cannot be excluded completely, and blood culture is certainly insensitive in the diagnosis of septicemia. Nevertheless, the majority of patients in the Vietnam series did not receive antibiotics during their hospital stay and recovered uneventfully. How then can this marked difference between the 2 studies be reconciled? The adult patients hospitalized in Myanmar with a diagnosis of malaria who were bacteremic had fever, neutrophil leukocytosis, and low malaria parasite counts [4, 5]. Indeed, their semiquantitative malaria parasite counts were significantly lower than in the nonbacteremic malaria patients, and they were orders of magnitude lower than the bacteremic patients in the Vietnam study. It is also unclear how many of the Myanmar patients fulfilled the WHO criteria for severe malaria [1]. In contrast, the patients in the Vietnam series all had strictly defined severe falciparum malaria, and concomitant bacteremias were associated with very high parasitemias, no neutrophil leukocytosis, and a high mortality [7]. The prevalence of bacteremia on admission was 0.65% (95% CI, .08% to 1.2%) in the Vietnamese patients with malaria parasitemias less than 20% (20% of erythrocytes containing one or more malaria parasites), which is 23 times lower than in the Myanmar series. 

The simplest and most plausible explanation for these marked differences is that the adult Myanmar patients with bacteremia did not have severe falciparum malaria but instead had bacterial sepsis as their primary illness, and they had incidental low density malaria parasitemia (a common finding in malaria endemic areas). Recent epidemiological studies across the Greater Mekong subregion, an area of low seasonal malaria transmission, show that asymptomatic parasite carriage in adults is more common than thought previously [6]. It is therefore not unexpected that adults living in these areas who develop bacterial infections might sometimes also have incidental low-density parasitemias. With the exception of enteric fever, neutrophil leukocytosis is another pointer to a primary bacterial infection. Emphasizing this diagnostic distinction is clearly of clinical importance. Thus, the current WHO guidelines that adults with severe falciparum malaria do not require empirical antibacterial drugs on admission seems reasonable based on current evidence. The one important exception is patients with very high parasite counts. In the Vietnamese patients with >20% malaria parasitemia, the prevalence of concomitant bacteremia was 5.2% (95% CI, .2% to 10.3%), a risk ratio of 8.1 (2.2 to 29.5). Whether 20% parasitemia is the optimum threshold for giving empirical broad spectrum antibiotics was not determined. This argues for rapid thin blood film assessment with accurate parasite counts in all patients suspected of having severe malaria [1, 7]. Severe malaria is a risk factor for bacterial infections, and there should be a low threshold for starting antibiotics in adults with severe falciparum malaria [1, 3], but those who are not hyperparasitemic do not require empirical antibiotics on admission.

## Notes

***Financial support.*** N. J. W. is a Wellcome Trust Principal Research Fellow.

***Potential conflicts of interest.*** The author: No reported conflicts of interest. The author has submitted the ICMJE Form for Disclosure of Potential Conflicts of Interest.
